# Origin of abnormal structural transformation in a (BiPb)FeO_3_/SrRuO_3_/SrTiO_3_ hetero-structure probed by Rutherford backscattering

**DOI:** 10.1038/s41598-017-04543-6

**Published:** 2017-07-03

**Authors:** Murtaza Bohra, Kartikeya Negi, Varun Karthik Y. S., Hsiung Chou, X. Wang, W. K. Chu

**Affiliations:** 1 Mahindra Ecole Centrale, Survey no: 62/1A, Bahadurpally Jeedimetla, Hyderabad, 500043 Telangana India; 2 Department of Physics, National Sun Yat-Sen University, Kaohsiung, 804 Taiwan; 3 Department of Physics and Texas Center for Superconductivity at University of Houston, Houston, TX 77204 USA; 4 Department of Nuclear Engineering, Texas A&M University, College Station, TX 77843 USA

## Abstract

Scientific efforts are growing to understand artificial BiFeO_3_/SrRuO_3_/SrTiO_3_-heterostructures, wherein an altered environment at each interface, caused by epitaxial strains, broken symmetry, off-stoichiometry and charge transfer, can generate a rich spectrum of exotic properties. Herein, (BiPb)FeO_3_/SrRuO_3_/SrTiO_3_-heterostructures were sputtered with various top (BiPb)FeO_3_-layers at different growth temperatures (*T*_g_). Strain relaxation at each interface changes with *T*_g_ and generates an additional peak alongside with (BiPb)FeO_3_ at a high *T*_*g*_ of 700 °C. Rutherford backscattering (RBS) was employed to understand this unusual behavior as to whether it is a mixture of two phases, layer splitting or inter-diffusion of elements. Surprisingly, complete overlapping of random and aligned RBS spectra from the sample with *T*_*g*_ = 700 °C indicates the presence of a large amount of defects/distortions at the interfaces. The RBS compositional analysis gives clear evidence of Fe and Ru vacancies to an extent that the structural integrity may not be maintained. This abnormal condition can be explained by the inter-diffusion of Pb and Bi elements into whole films and even into the top layer of the SrTiO_3_ substrate, which compensates for these vacancies by substitutional replacement and is responsible for the generation of the additional SrTi(BiPb)O_3_—peak. Below *T*_*c*_^SrRuO^_3_, the magnetic properties change significantly with *T*_*g*_.

## Introduction

Remarkable interfacial phenomena such as ferromagnetism at CaRuO_3_^paramagnetic^/CaMnO_3_^antiferromagnetic^, topological superconductivity at LaAlO_3_^insulator^/SrTiO_3_^insulator^ and the magneto-electric effect at La_0.5_Sr_0.5_MnO_3_^ferromagnetic^/BaTiO_3_^ferroelectric^ can provide a new avenue to realize artificial multifunctional materials which show physical properties that are not present in either of the constituent layer’s bulk materials^1–4^. Together, they have exposed a wealth of phenomena at the boundaries where compounds with different structural instabilities and electro-magnetic properties meet, providing unprecedented access to new physics emerging at oxide interfaces. After a report (Wang *et al*. ref. 5) of room temperature electric and magnetic polarizations in a BiFeO_3_^antiferromagnetic^/SrRuO_3_^paramagnetic^ heterostructure, there have been numerous studies identifying the origin of these enhanced properties as either epitaxial strain^5^ or doping induced altered BiFeO_3_ (BFO) crystal symmetries^6^. With recent advances in deposition techniques, the BiFeO_3_ heterostructure spans a wide range of crystalline structures and plays host to an incredible variety of physical phenomena. Recent studies show that using X-ray reciprocal mapping, neutron scattering and temperature-dependent scanning probe-based studies, the growth temperature, thickness and substrate symmetry-dependent evolution of complex BiFeO_3_ phases^7, 8^ range from distorted monoclinic (M_A_ and M_C_ phases) to tetragonal and eventually parent rhombohedral structure. However, the exact origin for these significant properties has remained elusive. Previously, our group has worked extensively with various SrRuO_3_^metal^/SrTiO_3_^insulator^, (BiPb)FeO_3_^antiferromagnetic^/SrRuO_3_^paramagnetic^ and (BiPb)FeO_3_^antiferromagnetic^/SrTiO_3_^diamagnetic^ heterostructures of interest^9–12^. Doping of Pb ion plays a vital role in stabilizing single phase BiFeO_3_ with weak ferromagnetic order while dramatically reducing leakages, which gives correlation between the processing crystal structure, magnetic properties and magnetoelectric couplings^8, 13^. In the recent study^9^, we used reciprocal space mappings (RSMs) to demonstrate structural expansion (from cubic to tetragonal, and finally a mixture of two tetragonal phases) and suppression of spiral spin state in Pb doped BiFeO_3_ ((BiPb)FeO_3_) layers with varying growth temperature (650–700 °C). However, results of electron paramagnetic resonance study are unclear in terms of whether the other secondary tetragonal structure observed in RSM is due to (BiPb)FeO_3_ or is due to the presence of some defects or other nonmagnetic impurity. In another study^11^, we have shown an inverse effect in (BiPb)FeO_3_/SrRuO_3_/SrTiO_3_ heterostructures, with an increase in thickness of the top (BiPb)FeO_3_layers (50–400 nm), and an anomalous decrease in unit cell volume of the bottom SrRuO_3_ (fixed thickness ~100 nm) layer. Usually, the properties of the subsequently grown top layers of heterostructures are affected by the interface formed between it and the preceding layer.

In order to comprehend these altered properties and new (BiPb)FeO_3_ phases, in-depth knowledge of interfacial phenomena of heterostructures is required before discovering devices. Although high-resolution X-ray reciprocal maps, transmission electron microscopy (HRTEM) and Raman spectroscopy have been the most preeminent techniques to study structural properties in terms of their lattices, it is generally difficult to apply the technique at an elemental level. In literature, commonly the best BiFeO_3_ films were obtained in the growth temperature range 600–700 °C either by PLD or sputtering^14–16^, and therefore it’s imperative to study these films at elemental level in order to get in depth knowledge of interfacial mixing or inter-diffusion of elements otherwise their stoichiometry will be remained doubtful. In most of the cases RSM and HRTEM gives crystal lattices which may lead to a wrong conclusion about exact crystal structures, because of the various nearly similar (BiPb)FeO_3_ crystal symmetries and twining present in the films. Here, we highlight new interface phenomena in (BiPb)FeO_3_/SrRuO_3_/SrTiO_3_ heterostructures grown at critical growth temperature range 650–700 °C by using Rutherford backscattering as a probe to identify the phases of each layers. We further propose a mechanism for the observed secondary phase that provides insight into the competing nature of the phases in this system.

## Results

**Figure 1:**
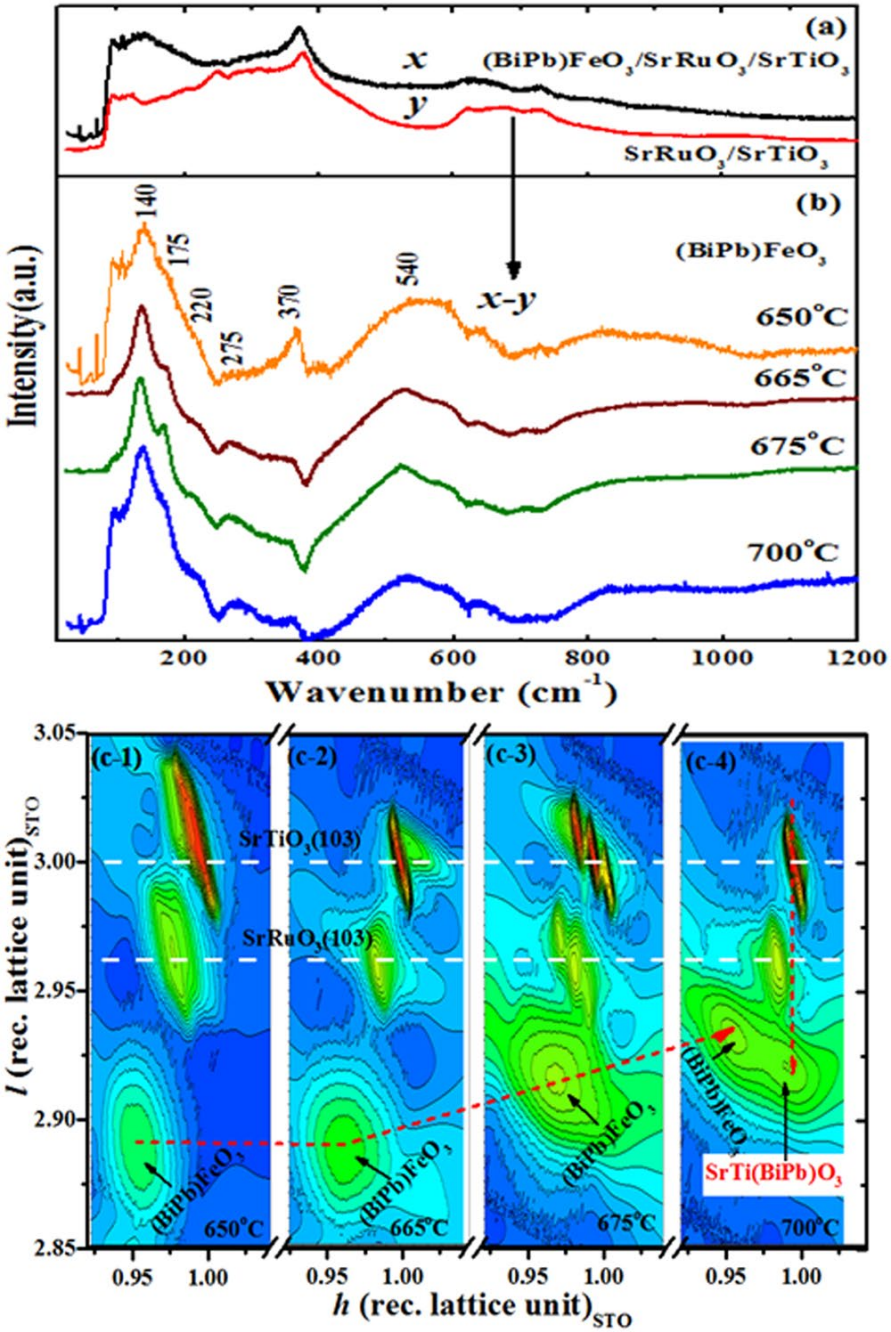
Raman spectroscopy and high resolution X-ray reciprocal space mapping of (BiPb)FeO_3_/SrRuO_3_/SrTiO_3_ hetero-structures. (**a**) Raman spectra of (BiPb)FeO_3_/SrRuO_3_/SrTiO_3_ hetero-structure for *T*_*g*_ = 650 °C (*x*) and the bottom SrRuO_3_/SrTiO_3_ structure (*y*). (**b**) Raman spectra represent only contributions from (BiPb)FeO_3_ layers (*x-y*) at different *T*_*g*_, showing only a few Raman modes when compared to bulk BiFeO_3_. (c: 1–4) RSMs around (103) planes of (BiPb)FeO_3_ layers^9^ and bottom SrRuO_3_/SrTiO_3_ structure; with increasing *T*_*g*_, the (BiPb)FeO_3_ peak splits into two peaks (indicated by vertical red arrow).

The (BiPb)FeO_3_ layers were grown on SrRuO_3_ coated SrTiO_3_(100) substrates at growth temperature, *T*_*g*_ = 650–700 °C, by a sputtering system^9, 12^. While Raman scattering has been employed to investigate the phase transformation and soft modes in the bulk BiFeO_3_, Raman spectra from (BiPb)FeO_3_ layers are in general difficult to obtain since an underlying perovskite SrRuO_3_/SrTiO_3_ structure overwhelms the scattering signal emanating from the top (BiPb)FeO_3_ layers. By subtracting the contributions arising solely from the SrRuO_3_/SrTiO_3_ structure, it is possible to obtain the contribution of the (BiPb)FeO_3_ layer in the Raman signal^17^. Figure 1a shows one of the representative Raman spectra of the (BiPb)FeO_3_/SrRuO_3_/SrTiO_3_ heterostructure grown at *T*_*g*_ = 650 °C (*x*) as well as the base SrRuO_3_/SrTiO_3_ (*y*) structure. Figure 1b shows the difference in spectra between curves *x* and *y* for the (BiPb)FeO_3_ layer (*x*-*y*). Applying this same method, the Raman spectra of other (BiPb)FeO_3_ layers grown at *T*_*g*_ of 665–700 °C are given in the same figure. These Raman spectra show prominent bands at 140.2, 175, 370.4, and 540.3 cm^−1^ slightly higher than rhombohedral bulk BiFeO_3_ energy values^18^, which correspond to BiFeO_3_ modes of A_1_(1TO), A_1_(2TO),E(TO) and E(TO), respectively. This shift in Raman bands can be ascribed to the Pb doping which may create minor changes in chemical bonds that surround the dopants. This also results in fewer number of Raman mode compared to the 13 Raman-active modes for bulk R3c rhombohedral BiFeO_3_ symmetry^18^. Although the fewer Raman modes in our (BiPb)FeO_3_ layers may reflect a space group with higher symmetry than the bulk R3c, we can still compare the spectra qualitatively to obtain some insight into (BiPb)FeO_3_ symmetry change with *T*_*g*_. The Raman spectra of the *T*_*g*_ = 650 °C (BiPb)FeO_3_ layer is slightly different from the other 665–700 °C layers. As *T*_*g*_ increases, small variances in Raman spectra are visible between the (BiPb)FeO_3_ layers. Indeed, in *T*_*g*_ = 665 °C films, the 175 cm^−1^ band starts appearing, and is completely developed in the *T*_*g*_ = 675 °C films, similar to bulk R3c BiFeO_3_. At high *T*_*g*_ of 700 °C, these two 140 and 175 cm^−1^ bands overlap again and become broad peaks, which gives an indication of mixing of phases or inter-diffusion of elements. The “pre-edge” before 140 cm^−1^ for 650 and 700 °C films is a cut-off result for the filter to avoid the tail effect of original incident beam.

Any changes in crystal symmetry due to applied strains caused by the substrate or a Pb doping-related effect results in either a shift in the frequency of Raman modes or a merging of Raman modes. For this purpose, X-ray RSMs can be an optimal tool to compute exact crystal lattices. The RSM images of (BiPb)FeO_3_/SrRuO_3_/SrTiO_3_ heterostructures grown at different *T*_*g*_, collected around asymmetric (103) SrTiO_3_ planes is shown^9^ in Fig. c1–4. All topmost (BiPb)FeO_3_ layers exhibit large in-plane lattices (*a* = 4.08–3.99 Å) compared to the SrRuO_3_ (*a* = 3.926 Å) and SrTiO_3_ (*a* = 3.905 Å), as shown in Table 1. In-and out-of-plane lattices of SrRuO_3_ layers are almost independent of *T*_*g*_. The strain relaxation, i.e., (*a* ^*BiPbFeO*^_*3*_ – *a* ^*SrTiO*^_*3*_)*/a* ^*SrTiO*^_*3*_, in (BiPb)FeO_3_ peaks (indicated by red bended dashed arrow) decreases with *T*_*g*_ from 650 °C (~5%) to 700 °C (~0.5%), approaching R3c bulk BiFeO_3_ symmetry, as is shown in the Raman spectra. For the *T*_*g*_ = 675 °C sample, one can see three SrTiO_3_ substrate peaks located at the (103) plane position, which simply indicates a substrate imperfection that propagates deeper into the (BiPb)FeO_3_/SrRuO_3_ structure. Surprisingly, at a high *T*_*g*_ of 700 °C, the films exhibit one new peak adjacent to the main (BiPb)FeO_3_ peak and exactly beneath the SrTiO_3_ substrate peak (indicated by vertical red dashed arrow). Raman data of this film also show an unusual merger of three 140, 220 and 275 cm^−1 ^bands. At this stage, we are uncertain whether these are mixed phases of BiFeO_3_ and_/_or splitting of the BiFeO_3_ layer or impurity phases. However, this case differs from Kim *et al*.^14^, where splitting of BiFeO_3_ peaks was observed when thickness increased from 70 nm to 980 nm, which resulted in the transformation of the tetragonal phase to the original R3c BiFeO_3_ phase. Another study by Bea *et al*.^15^ also shows the splitting of BiFeO_3_ peaks with an increase in thicknesses from 70 nm to 240 nm which were attributed to a monoclinic-to-tetragonal phase transformation. However, their results strictly cannot be compared to ours, because neither study changed the growth temperature nor used Pb doping. Hence, since using only Raman modes and lattice parameters is insufficient to determine the exact stoichiometry of (BiPb)FeO_3_/SrRuO_3_/SrTiO_3_ heterostructures, some local probe is necessary to investigate the elemental level deficiency/vacancy of the selected materials.

**Table 1:** Composition and thickness (*t*) of each layers of (BiPb)FeO_3_/SrRuO_3_/SrTiO_3_ hetero-structures deduced from simulated RBS spectra at different *T*_*g*._ Lattices parameters (*a* and *c*) estimated by RSM are also given in the Table.

*T*_*s*_ = 650 °C	*T*_*s*_ = 665 °C	*T*_*s*_ = 675 °C	*T*_*s*_ = 700 °C
(BiPb)_1_Fe_1.11_O_3±δ_ (*a* = 4.089, *c* = 4.057 Å; *t* = 65 nm)/Sr_1_Ru_0.86_O_3±δ_(BiPb)_0.018_ (*a* = 3.972, *c* = 3.952 Å; *t* = 125 nm)/Sr_1_Ti_1_O_3±δ_(BiPb)_0.02_(*t* = 35 nm)/Sr_1_Ti_1_O_3_ (*a* = 3.905 Å)	(BiPb)_1_Fe_0.93_O_3±δ_ (*a* = 4.066, *c* = 4.052 Å; *t* = 275 nm)/Sr_1_Ru_0.78_O_3±δ_ (BiPb)_0.022_ (*a* = 3.960, *c* = 3.957 Å; *t* = 112 nm)/Sr_1_Ti_1.05_O_3±δ_ (BiPb)_0.06_(*t* = 65 nm)/Sr_1_Ti_1_O_3_(*a* = 3.905 Å)	(BiPb)_1_ Fe_0.77_O_3±δ_ (*a* = 3.995, *c* = 4.019 Å; *t* = 265 nm)/Sr_1_Ru_0.65_O_3±δ_ (BiPb)_0.017_ (*a* = 3.958, *c* = 3.958 Å; *t* = 105 nm)/Sr_1_Ti_0.91_O_3±δ_ (BiPb)_0.055_(*t* = 88 nm)/Sr_1_Ti_1_O_3_(*a* = 3.905 Å)	(BiPb)_1_Fe_0.65_O_3±δ_ (*a* = 4.062, *c* = 3.944 Å; *t* = 310 nm)/Sr_1_Ru_0.73_O_3±δ_(BiPb)_0.14_ (*a* = 3.958, *c* = 3.957 Å; *t* = 101 nm)/Sr_1_Ti_0.65_O_3±δ_(BiPb)_0.087_ (*a* = 3.944, *c* = 4.014 Å; *t* = 20 nm)/Sr_1_Ti_1_O_3_(*a* = 3.905 Å)

**Figure 2:**
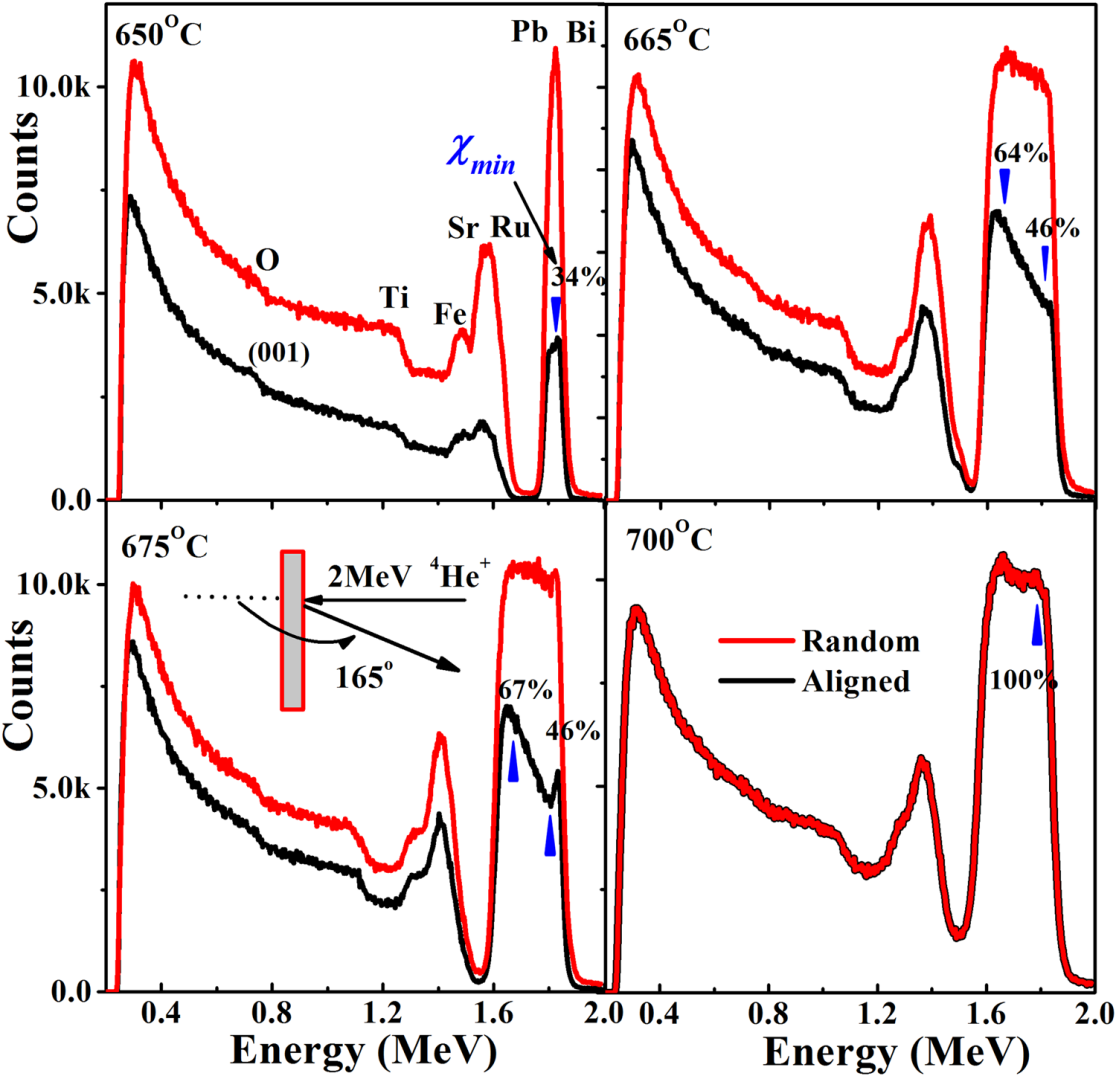
Random and aligned RBS spectra for (BiPb)FeO_3_/SrRuO_3_/SrTiO_3_ heterostructures. Random (red) and aligned RBS spectra (along [001] direction) (black) for (BiPb)FeO_3_/SrRuO_3_/SrTiO_3_ heterostructures. RBS ratio (*χ*_*min*_: 34 to 100%) (blue triangle) at (BiPb)FeO_3_ surfaces clearly indicates crystalline quality degradation and eventually completes an overlap of aligned to random spectra at high *T*_*g*_. Inset shows RBS experimental sketch.

We rigorously deployed Rutherford backscattering spectra for random and aligned cases on (BiPb)FeO_3_/SrRuO_3_/SrTiO_3_ heterostructures and the buffer layer SrRuO_3_/SrTiO_3_. RBS data was first analyzed qualitatively and then quantitatively. Figure 2 shows Rutherford backscattering (RBS) spectra taken in random and aligned modes. The leading edges from the Bi/Pb and Sr/Ru and Fe atoms in the films and those from the Sr, Ti and O atoms in the substrate are clearly seen. As shown in Fig. 2, Rutherford backscattering ratios (*χ*_*min*_ = 34 to 100%) yielded from aligned to random spectra near surface regions of top (BiPb)FeO_3_ layers (blue triangle). It indicates the degradation of crystalline quality with increasing *T*_*g*_. At *T*_*g*_ = 650 °C, the count of the aligned spectra of (BiPb)FeO_3_ slowly increases up to the top surface region of *χ*_*min*_ = 34%, indicating a possible presence of Bi/Pb and Ru vacancies. As *T*_*g*_ increases to 665 °C, a significantly large count is observed at the (BiPb)FeO_3_/SrRuO_3_ interface (*χ*_*min*_ = 64%), which decreases gradually to the top surface value (*χ*_*min*_ = 46%). A higher density of defects at the (BiPb)FeO_3_/SrRuO_3_ interface is mainly due to the additional lattice misfit strain apart from vacancies, which tends to become lower towards the top (BiPb)FeO_3_ surface, similar to the conventional strain-relaxation process^19^. Furthermore, at *T*_*g*_ = 675 °C, the count of *χ*_*min*_ = 67% increases in the sample and can be preliminarily explained as the introduction of extra crystal imperfections into the SrTiO_3_ substrate (as depicted in Fig. 1c). Surprisingly, at high *T*_*g*_ = 700 °C, we do not see any difference between random and aligned spectra, i.e., *χ*_*min*_ = 100%, which implies the presence of a large amount of defects/distortions/inter-diffusion which can enhance maximum backscattering even in (001) aligned configurations. We can also see the gradual diminishing of Fe peaks with increasing *T*_*g*_, indicating the presence of Fe vacancies in the (BiPb)FeO_3_ layer.

## Discussion

**Figure 3:**
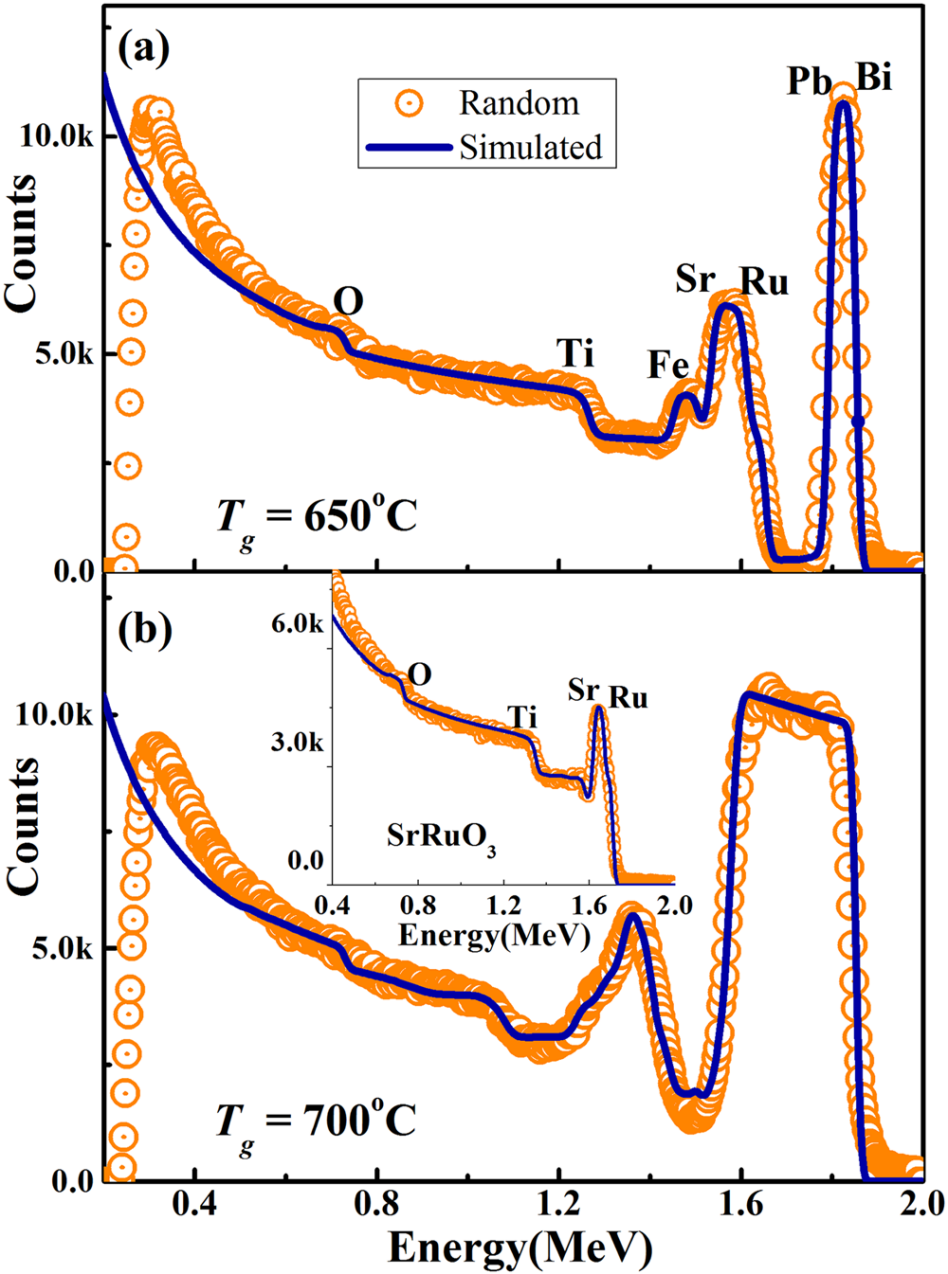
Elemental analysis of each layer of (BiPb)FeO_3_/SrRuO_3_/SrTiO_3_ heterostructures and bottom SrRuO_3_/SrTiO_3_ structure. RBS simulated data (*solid lines*) for (BiPb)FeO_3_/SrRuO_3_/SrTiO_3_ heterostructures of (a) *T*_*g*_ = 650 °C and (b) *T*_*g*_ = 700 °C are in good agreement with random data *(symbols)*. Variation in different edges of Pb, Bi and Fe indicate composition and thickness changes with *T*_*g*_. Inset of Fig. 3(b): Random and simulated spectra of bottom SrRuO_3_/SrTiO_3_ structure with Ru vacancies at surface of SrRuO_3_ layer.

**Figure 4:**
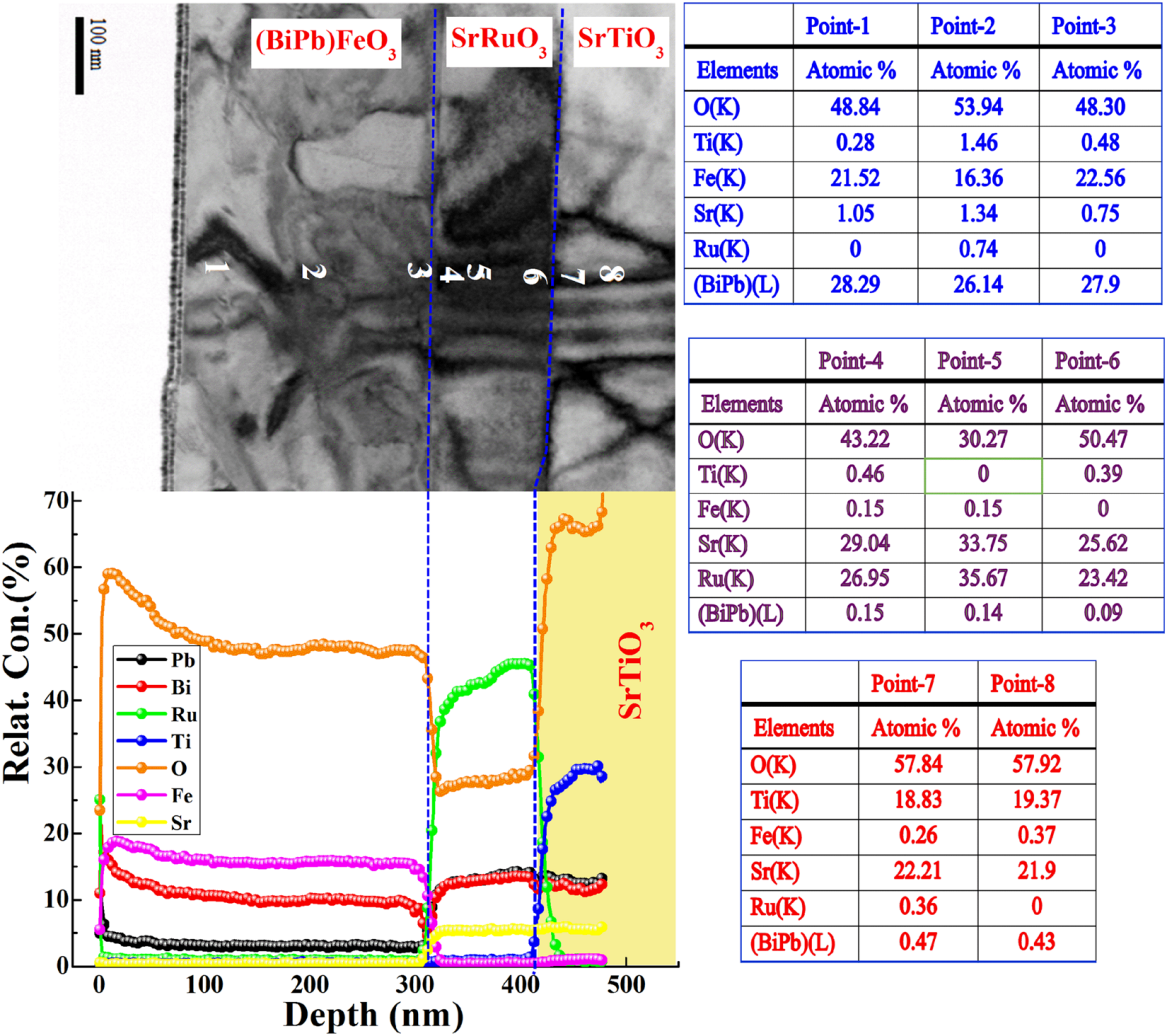
Compositional analysis of (BiPb)FeO_3_/SrRuO_3_/SrTiO_3_ heterostructures. Top left: STEM bright field image of (BiPb)FeO_3_/SrRuO_3_/SrTiO_3_ heterostructure with *T*_*g*_ = 700 °C, Bottom Left: Auger electron spectroscopy depth profiles with colour assigned to the different elements throughout the (BiPb)FeO_3_/SrRuO_3_ and SrRuO_3_/SrTiO_3_ interfaces^11^. This gives clear evidence of Bi and Pb inter-diffusion. Right: TEM EDS analysis across the points 1 to 8 shown on the bright field image.

In order to perform quantitative elemental analysis, the random RBS data for (BiPb)FeO_3_/SrRuO_3_/SrTiO_3_ heterostructures were simulated by using the Simulation Program (SIMNRA)^20^, and two of the representative sample plots (*T*_*g*_ of 650 and 700 °C) are given in Fig. 3. These random and simulated curves are in good agreement. The accurate determination of the Bi and Pb concentrations independently is difficult, because the contribution of Bi and Pb atoms to the RBS spectrum cannot be distinguished from each other. For convenience, the (Bi,Pb)_x_ concentration will be referred here as Bi concentration. The compositional analysis and thickness of each layers of (BiPb)FeO_3_/SrRuO_3_/SrTiO_3_ heterostructures from simulated spectra, as shown in Table 1, suggests four important features. First, we observe a severe deficiency of Fe element in top (BiPb)Fe_1−δ_O_3±δ_ layers with increasing *T*_*g*_. The Fe deficiency increases rapidly from 7% to 23% for films grown at 665 °C and 675 °C, respectively, and then increases further up to 35% for 700 °C films. Obviously, the extremely high deficiency cannot support a stable structure unless some other ions, such as Bi, Pb or O, can move to fill in the Fe ion vacancies. However, these high *T*_*g*_ films retain a crystalline structure with slightly smaller lattices compared to other films. Second, Ru vacancies in the bottom SrRuO_3_ layer, similar to the sample without the top (BiPb)FeO_3_ layer (shown in the inset of Fig. 3b), can be clearly observed. The Ru vacancy concentrations in all films are as high as Fe deficiency. Third, their structural integrity is maintained due to the interdiffusion of (BiPb) in the layer as well as the excess of oxygen filling in the Ru vacancies. The oxygen is the lightest element in the compound and is very difficult to quantify. Its concentration can be easily altered automatically in order to accommodate for any deficiency or vacancies of other elements. Instead of listing the exact quantities of oxygen, we use 3 ± δ to indicate its complexity. Interestingly, the Bi and Pb element inter-diffuses through the SrRuO_3_ layer and then penetrates even further into SrTiO_3_ substrates with *T*_*g*_ to form a very thin interlayer at the SrRuO_3_/SrTiO_3_ interface. For the 700 °C film, the interdiffusion of (BiPb) into the interlayer is estimated to be 0.087 mole. The fourth notable feature is that, along with the significant Ti vacancies, the interlayer of the 700 °C film obviously forms a crystal structure different from the pure SrTiO_3_ and becomes tetragonal with strained in-plane and relaxed out-of-plane lattices. This distinct interlayer contributes to the extra peak right below the SrTiO_3_ peak, as marked by a vertical dashed line, and by the SrTi(BiPb)O_3_ peak in Fig. 1c-4.

 ﻿Further support﻿ ﻿about Bi and Pb inter-diffusion into SrTiO_3_ substrate, we have performed TEM EDS analysis across the  (BiPb)FeO_3_/SrRuO_3_/SrTiO_3_ heterostructure with *T*_*g*_ of 700 °C, as shown in bright field STEM image (Top left Fig. 4). Compositional analysis Tables (Right Fig. 4) for each selected points from 1 to 8, are indicative that (BiPb)FeO_3_/SrRuO_3_/SrTiO_3_ heterostructure suffers Bi and Pb interdiffusion. On Auger electron depth profiling  spectra of (BiPb)FeO_3_/SrRuO_3_/SrTiO_3_ heterostructure (Bottom left Fig. 4), it demonstrates undoubtedly a significant interdiffusion of Bi and Pb elements at the (BiPb)FeO_3_/SrRuO_3_ and SrRuO_3_/SrTiO_3_ interfaces. But it is understood that TEM EDS shows only local reading while Auger  plot shows average reading of a large area of sample. However, the thicknesses (*x*-axis) in Auger plot was estimated using  SiO_2_  etching rate which may not take as a correct number. The inter-diffusion of Bi and Pb elements was also observed ^21^ in 0.7BiTiO_3_–0.3PbTiO_3_ films, when laser ablated on Pt/TiO_x_/SiO_2_/Si substrate. The Bi and Pb move through the entire porous Pt bottom electrode and sit just above the TiO_x_ adhesion layer. Although, SrRuO_3_ buffer layer was introduced  to prevent this interdiffusion between the substrate and the film, the Bi still appears to readily segregate from the other elements in the film towards the substrate. In our case, vacancies of Ru in SrRuO_3_ bottom layer facilities this inter-diffusion further deep into SrTiO_3_ substrate. With increasing (BiPb)FeO_3_ layer thickness^11^, the Bi and Pb inter-diffuses more rapidly into SrTiO_3_ substrate, which can be supported from fact that bottom SrRuO_3_ layer recovered their original bulk unit cell volume due to refilling of Ru vacancies by Bi and Pb elements. Therefore, it appears that inter-diffusion of Bi and Pb does take place, irrespective of method of deposition, however, inter-diffusion rate might be different like in RF-sputtering, sputter materials have higher kinetic energy compared to PLD and MBE processes. Thus, due to identical nature of Bi and Pb, both normally start inter-diffusion depending upon growth conditions and *in-situ/ex-situ* heat treatments, and the normal trigger point for inter-diffusion is above 600 °C. Study on interdiffusion (Bi and Pb) and vacancies (O and Ru) in the (BiPb)FeO_3_/SrRuO_3_/SrTiO_3_ heterostructure is crucial, as many novel properties like a switchable diode^22^ and photovoltaic^23^ effect and ferroelectric resistive switching^22^ phenomena can be tuned by controlling the oxygen vacancy migration. In the present (BiPb)FeO_3_ layers, more complicated phenomena are involved like interdiffusion, or doping, of Bi and Pb and the Fe vacancies. The former one, the doping of Pb, is responsible for generating O vacancies^24^ and must provide a very tight binding between them. For the later one (Fe vacancies), it can provide a path way for O vacancies to migrate under applied electric field. However, the migration of O vacancies will alter the fine balance of electricity and the polarization of (BiPb)FeO_3_ layer. Therefore, based on overall effects, a relatively higher driving force, or electric field, is expected to initialize the O vacancies migration. Our previous study^25^ indicating a subtle carrier accumulation at (BiPb)FeO_3_/SrRuO_3_ interface reflects a different band bending effect which causes the polarization vector of (BiPb)FeO_3_ pointing towards the interface at zero bias. How these different phenomena may affect the diode and photovoltaic effect are worthy to explore in near future.

**Figure 5:**
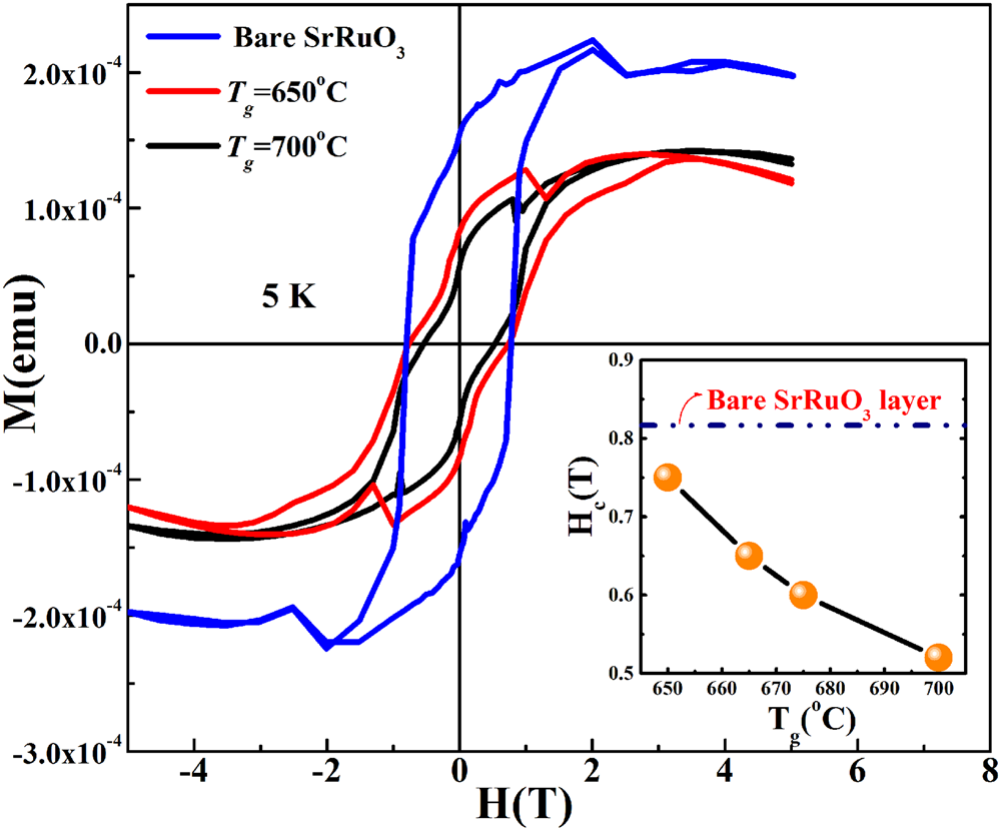
Low temperature magnetic properties of (BiPb)FeO_3_/SrRuO_3_/SrTiO_3_ heterostructures. *M*–*H* loops measured at 5 K in out–of–plane configuration for (BiPb)FeO_3_/SrRuO_3_/SrTiO_3_ heterostructures grown at *T*_*g*_ = 650 °C and *T*_*g*_ = 700 °C. Bare SrRuO_3_ layer *M*–*H* loop is also given for comparison. Inset shows decrease in coercivity *H*_*C*_ with *T*_*g*_ compared to the bare SrRuO_3_ layer.

The combined effects of inter-diffusion of (Pb and Bi) elements into SrRuO_3_ (which is paramagnetic at room temperature and ferromagnetic well below *T*_*c*_ of 150 K), as well as possible reduction in canted magnetism of (BiPb)FeO_3_ due to Fe vacancies, can be clearly seen in *M*–*H* loops (taken at 5 K) of (BiPb)FeO_3_/SrRuO_3_/SrTiO_3_ heterostructures grown at *T*_*g*_ = 650 °C and *T*_*g*_ = 700 °C, as shown in Fig. 5. The inset of Fig. 5 shows significant drop in the coercivity value, *H*_*C*_ for the *T*_*g*_ = 700 °C films compared to the *T*_*g*_ = 650 °C films as well as the *H*_*C*_ of the bare SrRuO_3_ layer around 0.2 T. Normally, the magnetization of both (BiPb)FeO_3_ and SrRuO_3_ layers are expected to decrease because of Fe and Ru vacancies in respective layers. However, presence of Bi and Pb may trap in part of Ru vacancies, and excess of Bi and Pb and Ru vacancies can distribute randomly in the SrRuO_3_ layer, creating local distortions that vary the angle and distance of Ru(site)–O–Ru(site) bonds and frustrate the long-range magnetic coupling^11, 12^. The Fe vacancy effect is more directly visible at 300 K, where SrRuO_3_ is in a paramagnetic state and the canted magnetic moment only arises from (BiPb)FeO_3_ being reduced drastically^9^ by 75% when *T*_*g*_ changes from 650 °C to 700 °C.

In summary, Rutherford backscattering spectroscopy  (RBS) is demonstrated to be a powerful technique for investigating the compositional analysis at the elemental level of each layer of (BiPb)FeO_3_/SrRuO_3_/SrTiO_3_ heterostructures. The RBS simulation analysis offers various recurring features, such as the formation of Fe and Ru vacancies in respective (BiPb) FeO_3_ and SrRuO_3_ layers, as well as robust inter-diffusion of Pb and Bi elements not only into bottom SrRuO_3_ layers but also deep into surface of SrTiO_3_ substrates. Beyond these, the inter-diffused element (Pb and Bi) coupled with Ti vacancies near SrTiO_3_ surfaces can modify the SrRuO_3_/SrTiO_3_ interface, which causes an additional thin inter layer of “SrTi(BiPb)O_3_”. This so-called thin layer of tetragonal crystal structure (similar to that of SrTiO_3_ substrate but with elongated *c-*axis) basically shows an additional peak in RSM, which is not a secondary phase of (BiPb)FeO_3_. This is a remarkable result, since, generally, observing the lattice without knowing the actual elemental compositions can mislead about the phases of same crystalline structure. These changes also drastically affect the magnetic properties of SrRuO_3_ and (BiPb)FeO_3_ layers.

## Methods

### Sample fabrication

The (BiPb)FeO_3_ layers were sputtered from a ceramic BiFeO_3_ (doped by 10% Pb) target at RF power of 200 W using a standard off-axis magnetron sputtering technique. Phase pure BiFeO_3_ (doped by 10% Pb) target was prepared by standard solid state reaction rout (shown in Supplementary Fig. S1 and Fig. S2). The (BiPb)FeO_3_ layers were grown at growth temperature, *T*_*g*_ = 650–700 °C under Ar/O_2_ (2/1) gas flow^9^. The bottom SrRuO_3_/SrTiO_3_(001) structure^12^, having in-plane lattices that match well with bulk BiFeO_3_, was used (grown at 690 °C, 200 W and Ar/O_2_ = 2/1) in order to minimize the strain effect on top (BiPb)FeO_3_ layers. SrTiO_3_(001) substrates were cleaned by the process described elsewhere^11^ prior to deposition. After deposition, the heterostructures were annealed under oxygen pressure of 250 Torr at 700 °C for 1.5 h to reduce possible oxygen deficiencies.

### Sample characterization

Raman spectra were recorded at room temperature by using a Jobin Yvon dispersion Raman microscope equipped with a CCD detector and a 16 mW He–Ne laser (633 nm) and were collected in backscattering configuration along the growth direction of the films. The spectra were measured in the range of 10–1200 cm^−1^ within an accuracy of 0.1 cm^−1^. It was confirmed that the laser power did not heat or modify the sample. Structural properties were measured by a Bede D1 high resolution X-ray diffractometer. Scanning transmission electron microscopy (STEM) images in bright field mode and energy dispersive x-ray spectroscopy (EDS) was performed with a field emission gun transmission electron microscope [(FE-TEM), FEI E.O Tecnai F20 G2] operated at 200 kV. An Auger electron spectroscopy depth profile was performed to analyze the composition of (BiPb)FeO_3_/SrRuO_3_/SrTiO_3_ heterostructure. The magnetic properties were measured with an out-of-plane configurations in superconducting quantum interference device. The diamagnetic contributions from the SrTiO_3_ substrate, glue/tapes and sample holder were subtracted from magnetization data by measuring the high-field magnetic susceptibility.

### Rutherford backscattering spectroscopy (RBS)

The compositional analysis of the (BiPb)FeO_3_/SrRuO_3_/SrTiO_3_heterostructure was carried out by Rutherford backscattering spectroscopy (RBS). A 2 MeV helium (4He^+^) beam was directed at the sample and the energy of the backscattered helium ions is measured at a back scattering angle of 165°. To minimize the possible charging effect at the top insulated (BiPb)FeO_3_ film, the sample were grounded by an C-tape. The RBS data analyses were performed by the simulation of the spectra using the Simulation Program^20^.

## Electronic supplementary material

Fig. S1 Crystalline structure determination of Pb doped BiFeO3. Fig. S2 Electron backscattering SEM images for BiFeO3 compound with and without doping of Pb.

## References

[1] Song C (2017). Recent progress in voltage control of magnetism: Materials, mechanisms, and performance. Progress in Materials Science.

[2] Grutter A J (2015). Electric field control of interfacial ferromagnetism in CaMnO_3_/CaRuO_3_ heterostructures. Phys. Rev. Lett..

[3] Mohanta N (2015). Inducing topological superconductivity at the LaAlO_3_/SrTiO_3_ interface. Journal of Physics: Conference Series.

[4] Cui B (2015). Manipulation of Electric Field Effect by Orbital Switch. Adv. Mater..

[5] Wang J (2003). Epitaxial BiFeO_3_ multiferroic thin film heterostructures. Science.

[6] Shelke V (2011). The role of SrRuO_3_bottom layer in strain relaxation of BiFeO_3_ thin films deposited on lattice mismatched substrates. J. Appl. Phys..

[7] Chen Z (2011). Low-symmetry monoclinic phases and polarization rotation path mediated by epitaxial strain in multiferroic BiFeO_3_thin films. Advanced Functional Materials.

[8] Wang J-S (2016). Evolution of structural distortion in BiFeO_3_ thin films probed by second-harmonic generation. Scientific Reports.

[9] Bohra M (2012). Structural expansion and suppression of spiral spin state in Pb-doped BiFeO_3_(00l) epitaxial thin films. J. Appl. Phys..

[10] Bohra M (2012). Strain relaxation in atomic flat SrRu_1-x_O_3_/SrTiO_3_ layers grown by off-axis RF-sputtering. IEEE Transactions on Magnetics.

[11] Bohra M (2012). Strain relaxation in Bi_0.9_Pb_0.1_FeO_3_/SrRuO_3_/SrTiO_3_heterostructures. J. Appl. Phys..

[12] Bohra M (2013). High symmetric SrRuO_3_(001) thin films: Perfectly lattice-matched electrodes for multiferroic BiFeO_3_. J. Appl. Phys..

[13] Mazumder R (2009). Effect of Pb-doping on dielectric properties of BiFeO_3_ ceramics. Journal of Alloys and Compounds.

[14] Kim DH (2008). Effect of epitaxial strain on ferroelectric polarization in multiferroic BiFeO_3_ films. Appl. Phys. Lett..

[15] Béa H (2007). Structural distortion and magnetism of BiFeO_3_epitaxial thin films: A raman spectroscopy and neutron diffraction study. Philosophical Magazine Letters.

[16] Wang Y (2017). Epitaxial growth of BiFeO_3_ films on TiN under layers by sputtering deposition. AIP Advances.

[17] Anooz SB (2010). Effects of post-growth annealing on physical properties of SrRuO_3_ thin film grown by MOCVD. Physica Status Solidi (A) Applications and Materials.

[18] Liu T (2011). Facile Route to the synthesis of BiFeO_3_ at low temperature. Journal of the American Ceramic Society.

[19] Chou H (2007). Particular strain relaxation for La_0.8_Ba_0.2_MnO_3_ films on SrTiO_3_(100) substrates. Appl. Phys. Lett..

[20] Mayer M (1999). SIMNRA a simulation program for the analysis of NRA, RBS and ERDA. AIP Conf. Proc..

[21] Bygrave, F. *et al*. Interdiffusion at the substrate-film interface of BiFeO_3_-PbTiO_3_ thin films on Pt/Si substrates. *IEEE International Symposium on the Applications of Ferroelectrics* (ISAF) (IEEE, New York, 2010), pp. 1–4.

[22] Wang C (2011). Switchable diode effect and ferroelectric resistive switching in epitaxial BiFeO_3_ thin films. Appl. Phys. Lett..

[23] Ge C (2016). Toward switchable photovoltaic effect via tailoring mobile oxygen vacancies in perovskite oxide films. ACS Appl. Mater. Interfaces.

[24] Chou H (2016). Oxygen deficiency-induced anomalous enhancement of Neel temperature and magnetic coupling for Bi_0.9_Ca_0.1_FeO_3-δ_ and Bi_0.9_Pb_0.1_FeO_3-δ_. Acta Materialia.

[25] Chou H (2013). Ferroelectricity of Bi_0.9_Pb_0.1_FeO_3_ films grown on atomic flat SrRuO_3_/SrTiO_3_ substrates. J. Appl. Phys..

