# Supplementary material for: Origin of abnormal structural transformation in a (BiPb)FeO3/SrRuO3/SrTiO3 hetero-structure probed by Rutherford backscattering
Source: Sci Rep. 2017 Jul 3;7:4501. doi: 10.1038/s41598-017-04543-6 (PMC5495773; doi:10.1038/s41598-017-04543-6)
Supplement: Supplementary file 1 — Fig. S1 Crystalline structure determination of Pb doped BiFeO3. Fig. S2 Electron backscattering SEM images for BiFeO3 compound with and without doping of Pb. [file 41598_2017_4543_MOESM1_ESM.doc]

**Supplementary Information**

## Origin of abnormal structural transformation in a *(BiPb)FeO_3_/SrRuO_3_/SrTiO_3_ hetero-structure probed by Rutherford* backscattering

Murtaza Bohra,^1, 2*^ Kartikeya Negi,^1^ Varun Karthik Y. S.,^1^ Hsiung Chou,^2*^ X. Wang, ^3, 4^ and W. K. Chu^3^

^1^Mahindra Ecole Centrale, Survey no: 62/1A, Bahadurpally Jeedimetla, Hyderabad-500043, Telangana India

^2^*Department of Physics, National Sun Yat-Sen University*, Kaohsiung-804, *Taiwan*

^3^Department of Physics and Texas Center for Superconductivity at University of Houston, TX 77204, USA

*^4^*Department of Nuclear Engineering, Texas A&M University, TX 77843, USA

*Corresponding author: [hchou@mail.nsysu.edu.tw (H.C.)](mailto:hchou@mail.nsysu.edu.tw (H.C.)); [murtaza@gmail.com (M.B.)](mailto:murtaza@gmail.com (M.B.))

**Supplementary Figures:**


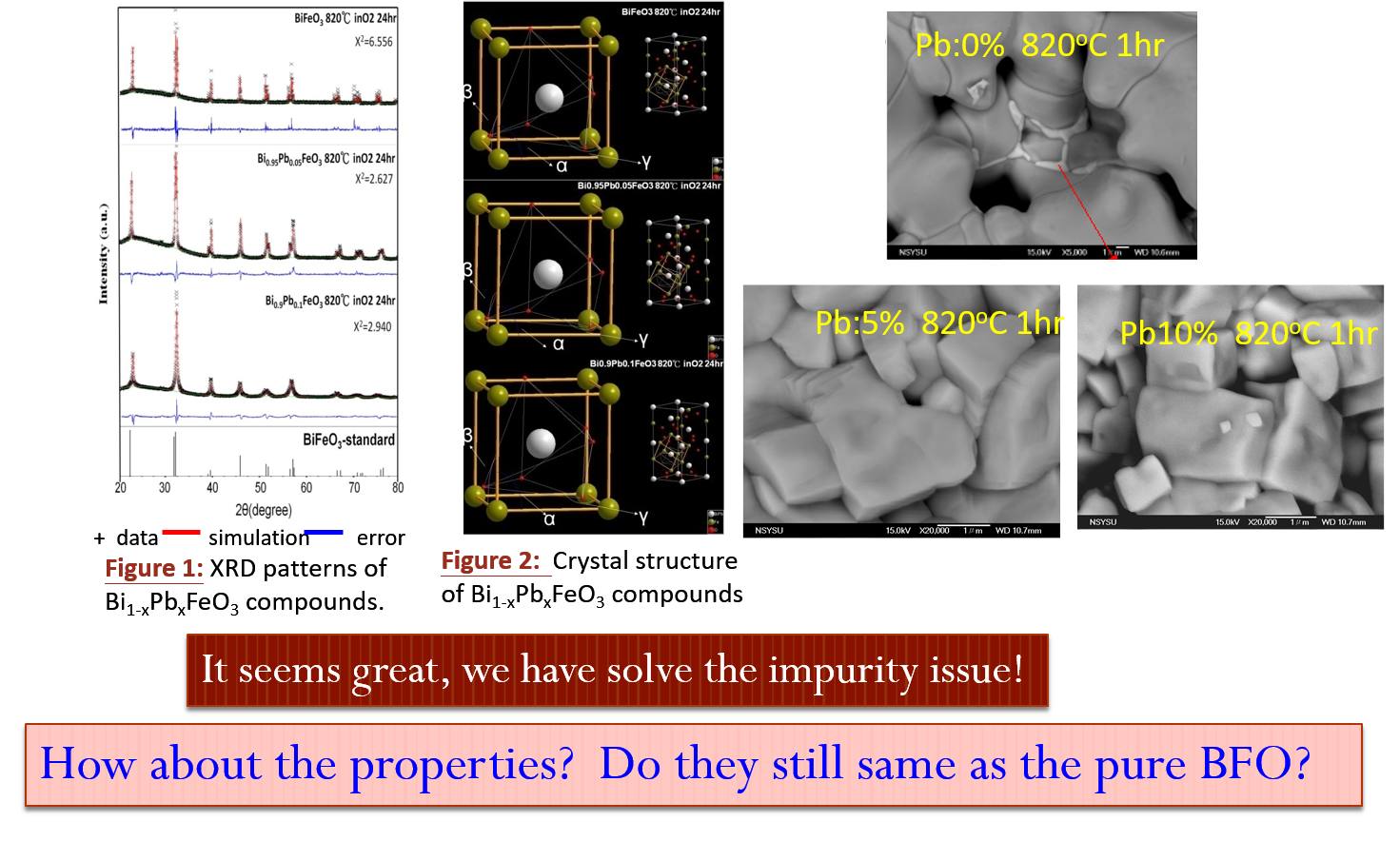


**Fig. S1 Crystalline structure determination of Pb doped BiFeO_3_. Left:** X-ray diffraction patterns (XRD) (by [Bruker D8 Advance](http://www.nano.nsysu.edu.tw/khvic/JL/E84.htm)) for Bi_1-x_Pb_x_FeO_3_(x = 0, 0.05 and 0.1) compounds prepared by ceramic rout. XRD patterns show the single phase Bi_1-x_Pb_x_FeO_3_ stabilizes after Pb doping. No impurity phase was traced. **Right:** Corresponding crystalline structures of BiFeO_3_ with and without doping of Pb.


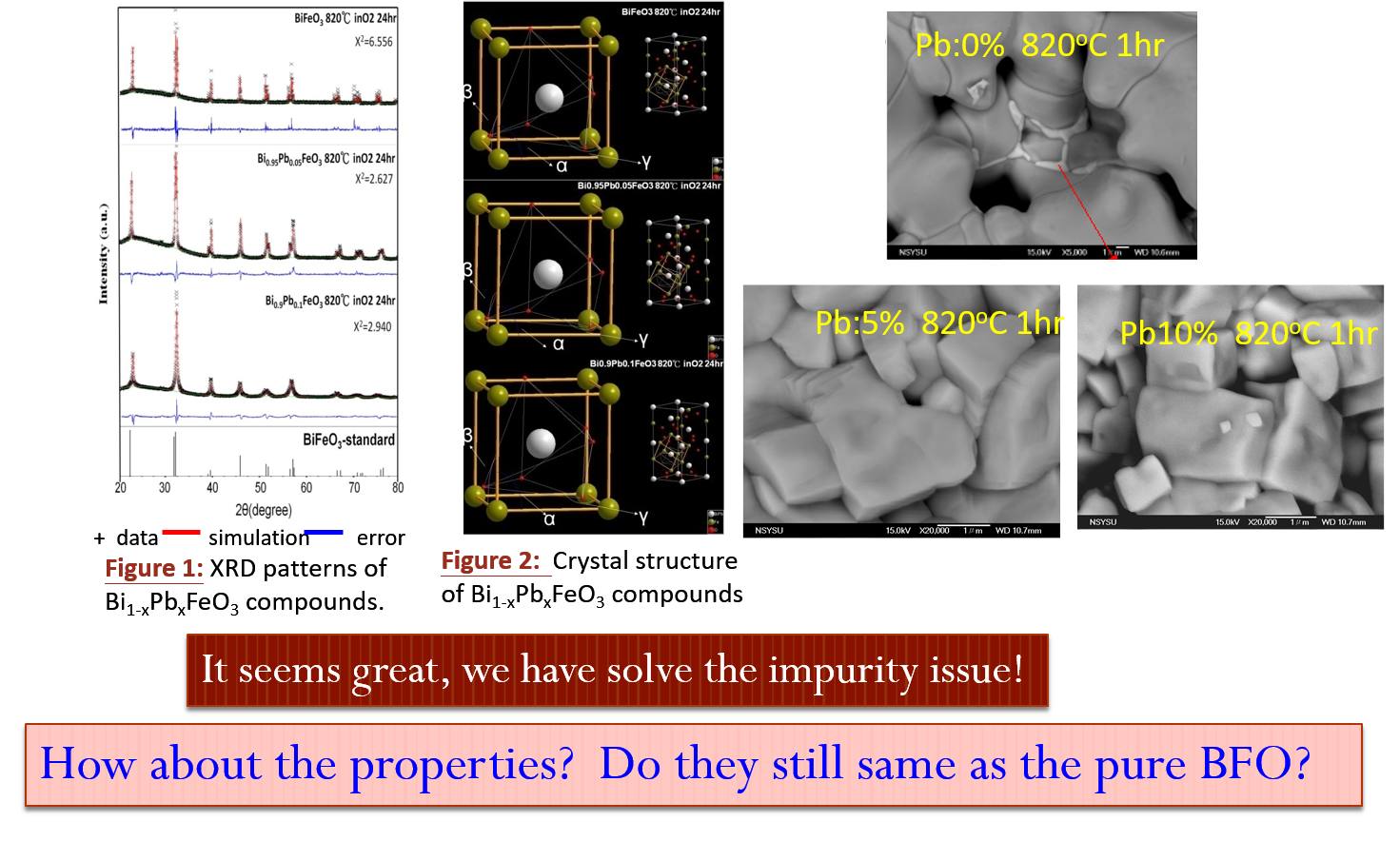


**Fig. S2 Electron backscattering SEM images for BiFeO_3_ compound with and without doping of Pb.** Backscattering Electron images (by [JEOL-6700 Field-Emission SEM/SEI/BEI](http://www.nano.nsysu.edu.tw/khvic/JL/E57.htm)) demonstrate that growth of BiFeO_3_ compound contains impurity phases trapped in between grain boundaries. However, after doping of Pb into BiFeO_3_, impurity phases can be suppressed drastically.
